# Physician Views on the Provision of Information on Immune Checkpoint Inhibitor Therapy to Patients with Cancer and Pre-Existing Autoimmune Disease: A Qualitative Study

**DOI:** 10.3390/cancers15102690

**Published:** 2023-05-10

**Authors:** Maria A. Lopez-Olivo, Gabrielle F. Duhon, Juan I. Ruiz, Mehmet Altan, Hussein Tawbi, Adi Diab, Clifton O. Bingham, Cassandra Calabrese, Natalia I. Heredia, Robert J. Volk, Maria E. Suarez-Almazor

**Affiliations:** 1 Department of Health Services Research, MD Anderson Cancer Center, The University of Texas, Houston, TX 77030, USA; gfduhon@mdanderson.org (G.F.D.); jiruiz1@mdanderson.org (J.I.R.); bvolk@mdanderson.org (R.J.V.); msalmazor@mdanderson.org (M.E.S.-A.); 2 Thoracic-Head & Neck Medical Oncology, MD Anderson Cancer Center, The University of Texas, Houston, TX 77030, USA; maltan@mdanderson.org; 3 Melanoma Medical Oncology, MD Anderson Cancer Center, The University of Texas, Houston, TX 77030, USA; htawbi@mdanderson.org (H.T.); adiab@mdanderson.org (A.D.); 4 Division of Rheumatology, Department of Medicine, Johns Hopkins University, Baltimore, MA 21205, USA; cbingha2@jhmi.edu; 5 Department of Rheumatologic and Immunologic Disease, Cleveland Clinic, Cleveland, OH 44195, USA; calabrc@ccf.org; 6 Department of Health Promotion and Behavioral Sciences, School of Public Health, Health Science Center, The University of Texas, Houston, TX 77030, USA; natalia.i.heredia@uth.tmc.edu; 7 Department of Internal Medicine, MD Anderson Cancer Center, The University of Texas, Houston, TX 77030, USA

**Keywords:** qualitative interviews, patient education, immunotherapy, autoimmune diseases

## Abstract

**Simple Summary:**

This study provides physicians’ perspectives on the information cancer patients with autoimmune diseases should learn when considering ICI. This information can be incorporated into patient–doctor discussions and educational tools to improve shared decision-making in this patient population.

**Abstract:**

Immune checkpoint inhibitors (ICIs) have improved cancer outcomes but can cause severe immune-related adverse events (irAEs) and flares of autoimmune conditions in cancer patients with pre-existing autoimmune disease. The objective of this study was to identify the information physicians perceived as most useful for these patients when discussing treatment initiation with ICIs. Twenty physicians at a cancer institution with experience in the treatment of irAEs were interviewed. Qualitative thematic analysis was performed to organize and interpret data. The physicians were 11 medical oncologists and 9 non-oncology specialists. The following themes were identified: (1) current methods used by physicians to provide information to patients and delivery options; (2) factors to make decisions about whether or not to start ICIs in patients who have cancer and pre-existing autoimmune conditions; (3) learning points for patients to understand; (4) preferences for the delivery of ICI information; and (5) barriers to the implementation of ICI information in clinics. Regarding points to discuss with patients, physicians agreed that the benefits of ICIs, the probability of irAEs, and risks of underlying autoimmune condition flares with the use of ICIs were most important. Non-oncologists were additionally concerned about how ICIs affect the autoimmune disease (e.g., impact on disease activity, need for changes in medications for the autoimmune disease, and monitoring of autoimmune conditions).

## 1. Introduction

The American Autoimmune Association has described more than 100 recognized autoimmune diseases [1]. More than 15% of the U.S. population suffers from autoimmune disorders (~50 million individuals, or one in five) [2,3,4]. Autoimmune diseases cause chronic inflammation that can alter DNA repair pathways [5]. Subsequently, DNA damage results in enhanced mutation frequency, cancer, and cell death [6]. Additionally, certain autoimmune disease treatments have been associated with increased cancer risk. A literature review revealed significant associations between more than 20 different autoimmune and chronic inflammatory autoimmune-related diseases with cancer [7].

Immune checkpoint inhibitors (ICIs) have been dramatically successful in treating various advanced solid tumors, including melanoma, lung cancer, and kidney cancer [8,9,10,11]. For cancer patients with autoimmune diseases, checkpoint inhibition is possible but requires careful monitoring because they are at higher risk for immune-related adverse events (irAEs) than cancer patients without autoimmune diseases [12,13]. Cancer patients with autoimmune diseases must make decisions about receiving ICIs with respect to potential survival benefits versus the risk of adverse events and autoimmune condition flares. Therefore, the objective of the present study was to identify the types of educational content perceived by physicians as most useful for cancer patients with pre-existing autoimmune diseases who are candidates to receive ICIs as well as the preferred delivery method for this content. Our findings provide key learning points for incorporation into patient–doctor discussions and educational tools.

## 2. Methods

The results of this study are reported according to the Standards for Reporting Qualitative Research [14].

### 2.1. Qualitative Approach and Research Paradigm

We used grounded theory methods to inductively identify and classify the information perceived by physicians to be of relevance for educational content. Our analytical methods were aligned with a social constructivist approach [15] because the physicians’ knowledge about the most relevant educational content was believed to be obtained through their interactions with their patients and their own work-related environment and experiences [16].

### 2.2. Researcher Characteristics and Reflexivity

Discussions with the physicians were led by an investigator with experience in cognitive interviews (M.A.L.-O.). The research team comprised clinical researchers with expertise in qualitative research (N.H., R.J.V., M.E.S.-A., M.A.L.-O.), knowledge synthesis (M.A.L.-O., M.E.S.-A., J.I.R.), patient education (R.J.V., M.E.S.-A., M.A.L.-O.), internal medicine (J.I.R.), rheumatology (M.E.S.-A., C.O.B., C.C.), and oncology (M.A., H.T., A.D.), with doctorate-level training in these areas.

### 2.3. Context and Sampling Strategy

We used convenience sampling and invited to participate all melanoma oncologists and thoracic/head and neck medical oncologists who had prescribed or were considering ICIs for their patients at The University of Texas MD Anderson Cancer Center and physicians in the departments of rheumatology, gastroenterology, and dermatology who had cared for or evaluated patients with pre-existing autoimmune diseases who were considering ICIs use. The size of the sample was largely determined by the availability of respondents and based on our previous research. We estimated that the recruitment of 15 to 20 physicians would achieve data saturation and allow us to fully explore in-depth each physician’s account [17,18].

### 2.4. Ethical Issues Pertaining to Human Subjects

This study was approved by the MD Anderson Institutional Review Board (protocol #2020-0035). A consent statement was provided to physicians, and any questions concerning the study were answered. Institutional standard procedures were followed to address data security issues.

### 2.5. Data Collection Methods

Eligible physicians were invited to participate with an email. The interviewer (M.A.L.-O.) had previous professional relationships (research collaboration) with three rheumatology physicians. The interviewer emailed each physician the day before each interview to remind them of the interview date and time and summarized the questions that would be asked. Participating physicians could opt to be interviewed in their clinics, on Zoom, or in their offices at the most convenient time from August to November 2021. Physicians who chose Zoom could turn their video off. Each session lasted about 45 min, and the audio was recorded.

### 2.6. Data Collection Instruments and Technologies

A semi-structured interview guide was created by the study authors (Supplementary Table S1). M.A.L.-O., developed the first draft of the guide and received feedback from the rest of the study team. Two pilot interviews were conducted to assess the interview length, check the flow, and confirm that the content accurately addressed the research questions. During the study interviews, our goal was to gain insight into the problems patients and providers experience when making treatment decisions. Our hope was that the data obtained would elucidate the approaches to decision-making physicians use for patients with autoimmune diseases and cancer and provide the most appropriate learning content to be delivered.

Information on the physicians’ sex, ethnicity, specialty, years of practice, and percent of time in the clinic was obtained using REDCap. We also asked how many of their patients receive ICIs per month on average and how confident they felt in managing patients with cancer and pre-existing autoimmune diseases who receive ICIs.

### 2.7. Data Processing

Interview recordings were transcribed verbatim using Adept Word Management (Houston, TX, USA). Transcripts were anonymized using identification codes. A research team member reviewed each interview and confirmed the transcript accuracy. Audio files were stored on a secured drive under an institutional server accessible by the research team only. Transcripts were then transferred to the web application Dedoose to code and analyze the data [19].

### 2.8. Data Analysis

A previously reported approach to thematic analysis was used [20]. Initial data familiarization was completed by M.A.L.-O. Next, independent coding of the transcripts was performed by 3 researchers (M.A.L.-O., J.I.R., G.F.D.). The transcripts were analyzed with a combination approach of deductive and inductive coding to list categories and subcategories of the data units (i.e., physicians’ statements and quotes for each question asked) according to the guiding questions to ensure our research objectives were met. M.A.L.-O. created a thematic map and checked themes against the data set before applying the themes’ meanings to the research question. A preliminary report included the identification and definition of themes (i.e., main categories of data) and subthemes (i.e., subcategories) emerging from the analysis. Saturation was considered when no new information was obtained from the data collected [21,22].

### 2.9. Techniques to Enhance Trustworthiness

To ensure study rigor and trustworthiness, the data were coded by different researchers. Interviews were listened to and compared against their transcripts. Three physicians were selected for a non-causal random institutional audit, and all standards for research without bias were met.

## 3. Results

### 3.1. Participants

We invited 24 physicians, but only 20 participated. Two-thirds were women. Six physicians were melanoma oncologists (30%), five were thoracic/head and neck medical oncologists (25%), four were rheumatologists (20%), three were dermatologists (15%), and two were gastroenterologists (10%). The years of practice ranged from 2 to 27. The average number of patients receiving ICIs seen per month ranged from 10 to 80. Most physicians felt confident in managing cancer patients with pre-existing autoimmune diseases receiving ICI (Table 1).

### 3.2. Synthesis and Interpretation of the Data

**Figure 1:**
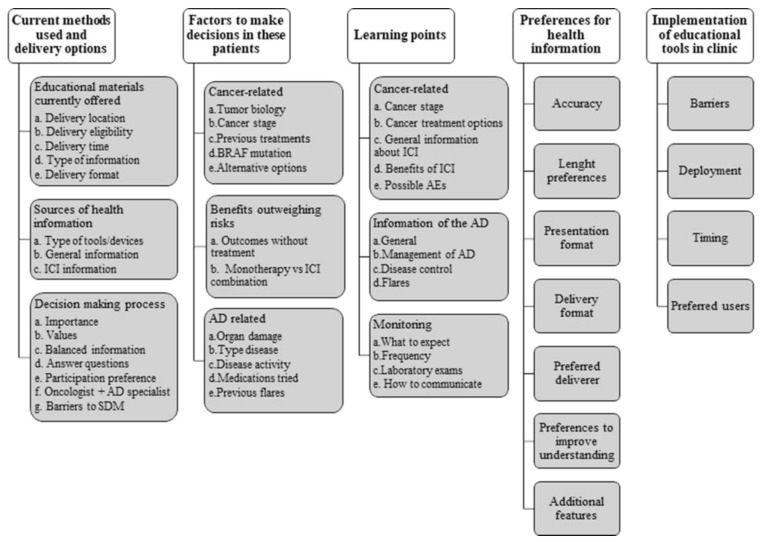
Themes and their subthemes identified in the physicians’ interviews. AD, autoimmune disease; AEs, adverse events; SDM, shared decision-making; ICI, immune checkpoint inhibitors.

We identified five themes from the interviews that aligned with our research objectives: current information provided, methods used, and delivery options; factors considered when making treatment decisions; key information to share with patients during patient–doctor discussions; preferences for optimal delivery of health information on ICIs for patients with cancer and pre-existing autoimmune diseases; and factors perceived as obstacles to and facilitators of the use of an educational tool or decision aid in this context (Figure 1).

#### 3.2.1. Current Information Provided (Methods Used and Delivery Options)

This theme had three subthemes: educational materials, perceived sources of health information used by patients, and factors involved in decision-making. Table 2 shows example quotes for salient subthemes.

- First, most physicians reported delivering information to patients in the examining room but not at every visit. All physicians expressed being unaware of any currently available materials specifically developed for patients with cancer and pre-existing autoimmune diseases. The available materials that physicians were aware of contain concise generic information about immune checkpoints inhibitors for all cancer patients. These materials are provided in-person by anyone available (in most cases, either the staff member obtaining patient consent for treatment or the physician) when patients consent to initiate therapy, and some patients receive pamphlets after discussions with their oncologists. Materials provided most often include handouts on drugs, materials developed in-house (by the institution), or materials offered by medical societies or organizations. Two physicians preferred drawing pictures of the information discussed with patients. Non-oncology specialists preferred to first learn about what was discussed with the oncologist to supplement the information already provided and more specifically address patients’ educational needs in the context of autoimmune disease. Most preferred to deliver information verbally and then send it through the electronic health record system (note with a summary of the discussion) for patients to review.
- For the perceived sources of health information used by patients, most physicians stated that most of their patients use electronic tools/devices to obtain health information, with Google and social media sites as the most common sources. Other common sources of information were the patients’ support groups (relatives, caregivers, friends, etc.) and cancer- and/or disease-specific societies.
- For the factors involved in decision-making, physicians described the methods used during decision-making for patients who are candidates to receive ICIs and are diagnosed with pre-existing autoimmune diseases. They said that shared decision-making is important to avoid decisional regret and emphasized first considering the patient’s values. All physicians also stressed the importance of presenting balanced information about benefits and risks, ensuring patients correctly interpret information, answering any questions (during or after the encounter), and accounting for patient preferences when making treatment decisions. In addition, most expressed the need to consider the decisions of patients’ support group members (e.g., family, caregivers, close friends) when patients want their involvement. Non-oncologists also mentioned the need for close communication with oncologists to facilitate decision-making and monitoring. Physicians listed several concerns regarding the decision-making process in this population. Shared decision-making was thought to require additional clinical personnel. Some participants thought patients may have anxiety when presented with the probability of flares or irAEs. Others mentioned insufficient time to cover all components of shared decision-making in visits, inability to complete a detailed electronic health record note summarizing the shared decision-making visit, and not having time to answer all questions or contact all interested parties in cases where the patient has a large support system.

#### 3.2.2. Factors to Make Treatment Decisions in These Patients

This theme also had three subthemes: factors associated with cancer, ensuring that treatment benefits outweigh the risks, and factors associated with autoimmune diseases.

- First, the cancer-associated factors to make treatment decisions in these patients were tumor biology (i.e., how effective ICIs are anticipated to be), cancer stage (i.e., metastatic or not), previous cancer treatments, availability of targeted therapies, and other alternative options.
- The second subtheme consisted of contemplating the consequences of autoimmune toxicity in the context of the survival benefit expected while considering the patient’s needs. Another item within this subtheme was the decision to use one ICI versus combination therapy owing to the higher probability of adverse events with a combination. Physicians reported accounting for patient frailty, autoimmune disease severity, and the specific effect of targeted inhibitors on different autoimmune diseases.
- Regarding autoimmune disease, physicians mentioned considering the type, disease activity, number of medications used for it, severity of previous flares, and organ damage.

#### 3.2.3. Key Information to Share with Patients

Information regarding cancer and ICIs was the first subtheme in this theme.

- The key points suggested were information on cancer stage, cancer treatment options, general information, and specific information about ICIs (i.e., mechanism of action, benefits/response rates/cancer progression, and probability of adverse events). Regarding possible adverse events, oncologists emphasized the probability of fatalities, symptoms to be aware of, the possibility of quality of life being affected or the need for hospice care, and the potential for pause or discontinuation of the ICI administration.
- Autoimmune disease information was the second subtheme. The key learning points centered on providing general information about the autoimmune disease (natural history of the patient’s autoimmune disease, emphasis on how patients differ), general management of the autoimmune disease, the importance of disease control (including steroid use), and risk of flares of autoimmune conditions with ICIs. Specifically, non-oncologists centered on how ICIs may affect the outcome of autoimmune disease: (1) impact on disease activity, (2) changes in medications for an autoimmune disease, (3) probability of flares of autoimmune conditions, (4) available treatment options for flares, (5) other possible irAEs, (6) symptoms requiring immediate attention, (7) follow-up and monitoring of autoimmune conditions, (8) good sources of information other than asking doctors, and (9) potential influence of steroids on tumor response to ICIs.
- The third subtheme was information about monitoring. Physicians emphasized the need to provide information on what to expect during and after treatment with ICIs, the expected frequency of visits to autoimmune disease physicians (preferred in-person), the importance of frequent laboratory exams, and maintaining close contact with providers, especially during the first three cycles of ICI administration.

#### 3.2.4. Preferences for Optimal Delivery of Health Information

All physicians expressed the need for an educational tool that can help patients be more aware of the factors when making treatment decisions and provide precise estimates of the benefits and risks of ICIs for those with pre-existing autoimmune diseases. Crucial requirements, as noted by nine physicians, were accuracy, simplicity (information should be presented concisely and graphically), and fixed information (as opposed to individualized or non-linear information).

Requirements also mentioned included multiple delivery formats (e.g., electronic medical record portal (MyChart) (with the possibility to add attachments), paper, videos (delivered by doctors or nurses), and websites (interaction with patients responding to questions)) with features for improving understanding (e.g., interaction with images, graphs, and tables), avoiding language barriers, accounting for literacy levels, and presenting basic information initially, with the possibility of selecting more in-depth information if desired to avoid overwhelming patients. Additional features suggested to improve comprehension of the information and facilitate decision-making were vignettes of patient–doctor conversations, patient stories, and online peer support groups (or possibly social media). Not all physicians favored including a risk calculator to provide personalized probabilities of adverse events because of validity concerns, the potential for patient misinterpretation of the probabilities, and uncertainty about the most appropriate outcomes to include. However, physicians who did favor this suggested creating a separate website to make a risk calculator available for physicians, mid-level providers, and trainees before encounters with patients.

#### 3.2.5. Preferences for Optimal Delivery of Health Information

Physicians mentioned the following barriers to using an educational tool in the clinic: the need for dissemination to make people aware and remind them that there is a tool available, the potential increase in consult time, and the possibility of over-alerting patients.

Most physicians suggested deploying tools in a format that will allow for maintaining their current workflows and ensuring the tools can be accessed within the electronic health record system or a separate website before, during, and after patient–doctor encounters. Few solutions to provide information while facilitating workflow were mentioned and included providing access to the information before the visit to let patients bring questions for the encounter with their doctors, letting a nurse discuss the information, use it only to determine patient preferences (patient averse to significant toxicities or bedridden and wants to be part of all treatment decisions, etc.), or to provide after-care or after-discussion summaries.

## 4. Discussion

We learned that educational materials specifically developed to meet the learning needs of cancer patients with underlying autoimmune diseases who are considering ICIs are lacking. Therefore, our study can serve as a comprehensive catalog of relevant information and delivery formats that, according to physicians, can facilitate decision-making for patients with cancer and pre-existing autoimmune diseases who are candidates to receive ICIs. Three main elements must be considered and understood by patients when discussing ICI initiation: cancer-associated characteristics that inform the probability of success/survival, autoimmune disease-associated characteristics that inform the probability of irAEs/flares, and the consequences of any potential treatment-related harms that may affect the patient’s quality of life.

To the best of our knowledge, the literature contains no studies exploring the most important educational topics about ICIs for patients with cancer and pre-existing autoimmune diseases according to physicians who treat these diseases. Previous qualitative and mixed methods studies focused on the perceptions of patients with specific types of cancer without pre-existing autoimmune diseases receiving ICIs or the needs of patients with irAEs [23,24,25,26,27,28,29,30,31,32,33,34].

A qualitative study by Fraterman et al. identified what patients should expect in regard to health information and technology applications [23]. In contrast with our physicians’ preference for a more generic approach to providing information, Fraterman and colleagues reported that inability to personalize educational tools and notifications was a barrier to patients’ use of technology applications. Furthermore, the key topics raised by patients in that study were similar to those raised by the physicians in our study, including clinical management and available supportive care services. However, a topic relevant to patients not mentioned by the physicians in our study was related to what patients can do to support their physical and mental well-being and symptom monitoring using mobile applications to facilitate patient–doctor communication and help patients feel more secure [23].

Another qualitative study reported the experiences of cancer survivors and their care needs after receiving ICIs [24]. In that study, patients also emphasized the need for more tailored health information, and similar to what we observed, they wanted information about how and when to communicate with their providers. Cappelli et al. also explored the needs of people with inflammatory arthritis induced by ICIs [27]. They highlighted the impact of irAEs in different domains of cancer patients’ quality of life and how these events influence the patients’ decision-making regarding the continuation of treatment. This aligns with the physicians’ statements in our study indicating that they discuss preferences about quality of life and ultimate treatment goals to avoid decisional regret with their patients. Researchers in two mixed methods studies also reported on this topic. One from Germany highlighted the need to increase patients’ general knowledge about ICIs and to increase risk awareness given that most patients perceived adverse events to be significantly less severe than those of other therapies [25]. A study from the United Kingdom also suggested creating content emphasizing the variability among patients when covering information about adverse events [26].

One qualitative study gathered physicians’ perspectives on emerging treatments for locally advanced unresectable or metastatic urothelial carcinoma [35]. The authors explored the factors that influence providers’ treatment recommendations. Like our findings, they observed that decision-making was influenced by the patient’s characteristics and comorbidities, tumor biology, and goals and preferences for treatment. It is important to note that the physicians also expressed the need for more general (as opposed to tailored) education, demonstrating potential discordance between patients’ and physicians’ perspectives regarding learning needs that can facilitate decision-making in the context of ICIs use.

The last theme explored the potential barriers to using educational material in the clinic with the potential increase in consult time being the one most mentioned. Most agreed that developing an educational tool for this population would be important, but few physicians provided solutions for implementing such a tool. Other studies have evaluated barriers and facilitators of educational tools in other medical contexts and, contrasting with our results, solutions provided include increasing the interest of physicians in the use of the tool, forming alliances between clinicians and researchers, and offering training in the use of the tool [36,37,38].

Our study has some limitations. Our findings are derived from interviews with physicians at a single U.S. comprehensive cancer center. Relevant elements identified for the development of educational tools may differ in other centers or countries. Furthermore, the physicians in our study may have had an interest in the development of educational tools. We did not explore potential barriers to health care such as insufficient or lack of health insurance, which could be another determining factor when making decisions for these patients, as patients seen at our institution for the most part are insured. Lack of health insurance coverage has been associated with poor receipt of cancer care in the U.S. [39]. Although we included physicians more likely to see patients with common autoimmune diseases who may receive ICI, such as inflammatory bowel disease, psoriasis, or rheumatoid arthritis, not all subspecialties could be represented. Providers in subspecialties not included in our study may have different perceptions about the key learning points or requirements for educational tools for their patients. Nonetheless, given that we used a semi-structured interview including open-ended questions and reached saturation after 13 providers, additional interviews would likely not change our conclusions. Furthermore, the investigators’ observations, intentions, and prejudices may have influenced the results. We tried to minimize this by avoiding any previous assumptions when analyzing the data and primarily using the natural language used by the physicians interviewed. Finally, we dealt with learning needs from the physicians’ perspective, and examining the views and opinions of patients in a similar qualitative study is necessary.

## 5. Conclusions

In conclusion, our findings have implications for the development and implementation of educational tools aimed at cancer patients with pre-existing autoimmune diseases considering treatment with ICIs. Our qualitative study provides important new information from the physicians’ perspectives on the information cancer patients with autoimmune diseases considering this treatment must learn. In this work, we gained insight into the current methods used to inform these patients and how the information is delivered, the factors that physicians consider when making treatment decisions regarding ICIs for these patients, the physicians’ most important learning points for developing educational content that can facilitate shared decision-making in this context, preferred methods of delivery of educational content in the clinic, and potential barriers to and facilitators for implementation. The next step after obtaining patients’ preferences for content and optimal ways to deliver it is to develop an educational tool incorporating our results. Our findings can also be used in patient–doctor discussions to improve shared decision-making in this patient population.

## Figures and Tables

**Table 1:** Characteristics of the participants (n = 20).

Characteristic	N (%)
Specialty	
Melanoma oncology	6 (30%)
Thoracic head and neck medical oncology	5 (25%)
Rheumatology	4 (20%)
Dermatology	3 (15%)
Gastroenterology	2 (10%)
Confidence in managing patients with cancer and pre-existing autoimmune diseases receiving immune checkpoint inhibitors	
Extremely	4 (20%)
Quite a bit	9 (45%)
Sex, Female	12 (60%)
Race and ethnicity	
Asian	10 (50%)
Non-Hispanic White	8 (40%)
Hispanic White	2 (10%)
Years of practice, mean (±SD)	11.5 (±11.1)
Percent clinical effort, mean (±SD)	47.5 (±22)
Number of patients receiving immune checkpoint inhibitors per month	53.4 (±50.5)

**Table 2:** Example of quotes for salient subthemes.

Subtheme	Physician Type	Example Quote
Delivery of educational materials currently offered	Oncologist	“*We want to document our discussion with the patient. So, there’s a smart phrase that we can do and say, ‘We’ve reviewed this following documentation’. So also, they [patients] can go back and look at it again in case they lose the paper, right? Because everybody can access their medical records.*”
Perceived sources of health information used by patients	Non-oncologist	“*Unfortunately, the internet seems to be popular in terms of like social media more and more these days. I get a lot of like follow-up questions about like,* “*Oh, I joined this Facebook group, and they said I should do this.*” *So, I guess that’s something that I see. Collective opinions online seem to drive a lot of information these days.*”
Factors involved in decision-making	Oncologist	“*Because of the seriousness of the consequences about—if they take it or not; it can go either way. And they need to know—I mean, they’re here, they have to give us preferences about quality of life, cancer treatment, the ultimate goal because this is—now, life and death...*”
	Non-oncologist	“*Anyhow, so we provide the information. We kind of talk through it. And then the inevitable, ‘what would you do if you were me?’ I kind of move that around a bit, and then, ‘what would you recommend?’ That’s something that I can give recommendations for, or they’ll say, ‘I’m leaning this way’, and I’d say ‘I think that that is a reasonable approach’. But for me, it’s giving them the information and then talking through it together with them to come up with a plan that both honors kind of their values and needs and is also medically sound.*”
	Non-oncologist	“*So, it’s always a shared decision—so my—oncologist discussion with the patient, and then my discussion with the patient, and then me and the oncologist—I always email this to the oncologist team with my recommendation, my impression. So, it’s going to be always through emails, and we all can decide so we can know what we are anticipating after starting the treatment.*”
Cancer-associated factors to make treatment decisions	Oncologist	“*…are there good, viable alternatives to immunotherapy for us specifically? That means does the patient have a BRAF mutation [for melanoma]? Would targeted therapy be a reasonable alternative with BRAF-directed targeted therapy again, either in adjuvant or the metastatic setting?*
Benefits of outweighing the risks	Oncologist	“*Think of it a little bit like a seesaw. On one side, you put things that are going to benefit the patient, and on one side, you put things that are going to cause harm to the patient. At the end, you sort of do this balancing act.*” “*Then you start quoting down to that particular patient. If, for instance, they are a violin player and make a living playing the violin, one of the side effects is peripheral neuropathy or impediment of nerves at a fine finger movement-- that’s important to them. If it was a young lady who is of reproductive age, and you have a risk of impeding that, that’s important to them. Those are different than talking to 85-year-old man or a woman who retired and is fairly sedentary and is definitely not in childbearing age. So first, the medical recommendation, the rationale behind it. Second, the general side effect profile, and third, the side effect profile as it relates to that particular individual.*”
Autoimmune disease-associated factors to make treatment decisions	Non-oncologist	“*Well, we’d like to know what therapy they’re on, obviously, the type of autoimmune disease that they’re receiving, the severity of the autoimmune disease, when their last flare was, and I suppose most importantly, actually, is—I guess we, most of all, need to know this beforehand—is what is the urgency and the indication for doing immune checkpoint therapy over other types of therapies. So, yeah, there’s a lot of questions that probably would give this more context.*”
	Oncologist	“*We sort of want to know how active their autoimmune disease is, whether they’re taking immuno—whether they’re requiring immunosuppression, certainly what type of autoimmune disease they have, and what are the other treatment options that are available for the patient.*”
Key cancer-related information to be provided	Oncologist	*Yeah, well, I mean, I think that the first thing is that probably with every patient, the part that they need to know first—many patients ask us—is, if I don’t do anything, what is my prognosis, or what’s the potential impact of not doing any treatment?* *And in the same way, really talking about what the efficacy and safety is—that we’ve seen in clinical trials is relevant for all of our patients. I think the part that we have that’s different for patients with preexisting autoimmune conditions is talking about where we do have gaps in our understanding because those patients really were largely excluded from those clinical trials. But in terms of, again, which parts are most important, it really does come down. There are some patients who come in once it be as aggressive as possible, regardless of what the risk is, and for those patients, certainly, they’re going to want to focus primarily on the efficacy data. But then, it’s absolutely important for them to understand what the risk data is—what the toxicity data is. We have other patients who come in really primarily focused on quality of life and being able to work in things like that, where for them, toxicity is a primary determinant of what therapy they receive over the efficacy data. And so—but again, that’s one where we still need to talk about both of those things. So, with all patients, I talk about both efficacy and toxicity. I don’t think I have any patients where I only talk about one or the other.*
Key autoimmune disease-related information to be provided	Oncologist	“*Well, I mean, usually if we’ve made a decision that we’ve recommended that the patient be treated with immunotherapy, we’ll do the informed consent where we go over again, sort of the standard information about all of the potential side effects that can happen, and then we will provide them in addition with this document that our team has generated for patients. Again, giving them sort of practical tips about what side effects to look out for, which ones can be managed themselves and how to manage them, but also what types of things should they really be calling their provider team for if those things start to happen. So commonly around sort of diarrhea, that type of stuff, we do not, to be honest, really talk about much more for patients who have a history of autoimmune disease, but it’s controlled. We really don’t come up with any different information for them. We really give them the same information that we get to all patients because of the fact that, yes, there is a risk of them exacerbating their preexisting condition. We also recognize that they could have completely new or different types of autoimmune effects that are different from their preexisting condition. So we still really go over everything with them, just like we do with patients without a history of autoimmune disease.*”
Key monitoring-related information to be provided	Oncologist	“*And they always have access to my clinic. They email me; they email my nurse—like I alert them which kind of symptoms they need to know about so they immediately can—(contact me).*”
Crucial requirements for optimal delivery of health information	Oncologist	“*I think just repeated, consistent, reliable education material. So, just pointing them to―(INSTITUTION) has a go-to with everything, where, with a click of a button― So if they have―if there is a resource that they are―they don’t have to look at many different web pages and websites and put multiple buttons, but they’re able to go to one site where they can enter their information and get all the necessary information they need to make an informed decision. I think that―that will help.*”
Barriers to using an educational tool in clinic	Oncologist	“*And sometimes, I don’t have time to even do a great job in really outlining all the discussion steps and the pros and the cons. I just say toxicity for this stuff, which I know is not optimal, but I’m working with constraints of time. So again, would it be nice to have? The answer is yes. Can I spend the extra time to do this? No. But would it be useful? I think very much so, right?*”
Solutions for deployment of an educational tool in clinic	Oncologist	“*So, if you embed it in EPIC [an electronic medical record system], it might not be easy to get at. If you have it as a separate website, maybe easier, or have EPIC point at a website. That’s another way of doing it…* *You know, I think we’re already sharing decisions with patients. We bring what we have to bring to make that decision. I don’t think it’s going to be a huge imposition to the workflow as long as it’s snappy and quick in EPIC.*”

## Data Availability

The data that support the findings of this study are available from the corresponding author, M.A.L.-O., upon reasonable request. The data are not publicly available due to containing information that could compromise the privacy of research participants.

## Supplementary Materials

The following supporting information can be downloaded at: https://www.mdpi.com/article/10.3390/cancers15102690/s1, Table S1: Semi-structured interview used.
