# Supplementary material for: Physician Views on the Provision of Information on Immune Checkpoint Inhibitor Therapy to Patients with Cancer and Pre-Existing Autoimmune Disease: A Qualitative Study
Source: Cancers (Basel). 2023 May 10;15(10):2690. doi: 10.3390/cancers15102690 (PMC10216836; doi:10.3390/cancers15102690)
Supplement: Supplementary file 1 [file cancers-15-02690-s001.zip › cancers-2341581-supplementary.pdf]

**Table S1.** Semi-structured interview used.

| Topic | Questions |
|---|---|
| Decision-making preferences | What factors are important to you when making decisions about whether to initiate immune checkpoint inhibitors in cancer patients who have a preexisting autoimmune disease?<br>What types of information do you give cancer patients with autoimmune disease when discussing treatment with checkpoint inhibitors?<br>Do you use any patient educational tools or aids during the medical encounter to present this information? |
| | What are your thoughts about shared decision making?<br>What is the role of family members or caregivers in the decision?<br>What are the main differences between using monotherapy (CTLA4, PD1/L1), and combination therapy in patients with autoimmune disease? |
| Informational needs | What do cancer patients with a preexisting autoimmune disease need to know in order to make an informed decision about whether to receive immune checkpoint inhibitors?<br>How would you explain and present information on the above factors to your patients?<br>Do you have any concerns about providing educational tools to your patients? |
| Format and delivery | Which delivery methods might be best to provide this type of information to patients? |
| Current materials or tools used | What types of information and educational materials on checkpoint inhibitors are currently available to your patients? |
| Implementation | What changes would be needed in your clinic to implement the use of patient decision aids? |
